# Psychophysical dissection of temporal error monitoring

**DOI:** 10.1007/s10339-025-01302-8

**Published:** 2025-09-30

**Authors:** Tutku Öztel, Fuat Balcı

**Affiliations:** 1 Department of Psychology, George Mason University, Fairfax, USA; 2 Department of Psychology, Koç University, Istanbul, Turkey; 3 Department of Biological Sciences, University of Manitoba, 50 Sifton Road, Winnipeg, MB R3T 2M5 Canada

**Keywords:** Error monitoring, Weber’s law, Time perception, Interval timing, Bayesian information criterion

## Abstract

The recent line of research robustly demonstrated that humans and rodents can keep track of the magnitude and direction of timing errors, composing a temporal error monitoring ability (TEM). However, the degree of dissociation between these two measures of TEM has not been investigated at the level of the underlying mental magnitude metrics. Specifically, we do not know whether the two behavioral manifestations of TEM differentially rely on subjective vs. objective time, whether the discriminability of time intervals relies on ratio and absolute differences, respectively. To this end, we first tested whether behavioral manifestations of TEM depend on relative (cognitive timing) or absolute timing errors (sensorimotor timing). In light of our earlier findings showing differential metacognitive processing of timing errors as a function of different levels of agency, we also tested whether the potential information processing differences in TEM measures differ across different levels of agency of timing errors? In two different datasets, we found that magnitude and direction monitoring of timing errors relied on the absolute (i.e., arithmetic/linear) and relative (i.e., ratio) distances, respectively. These effects were more pronounced for owned versus unowned errors for timing error magnitude monitoring and timing error direction monitoring, respectively. Together, this study demonstrated that the timing error direction monitoring relies more on cognitive timing, whereas error magnitude monitoring relies more on sensorimotor timing.

**Supplementary Information:**

The online version contains supplementary material available at 10.1007/s10339-025-01302-8.

## Psychophysical dissection of temporal error monitoring

In the simplest form, metacognition is defined as “cognition about cognition” (Flavell 1979). One critical aspect of metacognition is error monitoring, which refers to keeping track of one’s errors without external feedback (Yeung and Summerfield 2012). Recent studies have demonstrated that humans can keep track of the magnitude and direction of their temporal errors (temporal error monitoring e.g., Akdoğan & Balcı, 2024; for a detailed discussion, see: Öztel and Balcı 2024b). Further, one recent study demonstrated that magnitude and direction monitoring constitutes distinct components of temporal error monitoring ability, relying on different levels of agency (Öztel and Balcı 2024c). Given that time perception relies on Weber’s Law (which posits that discriminability of stimulus intensities relies on their relative ratio instead of absolute difference; e.g., Eijkman et al. 1966), one pending question is whether distinct components of temporal error monitoring ability differentially abide Weber’s law.

The ability to monitor errors has mostly been investigated in the perceptual and memory domains. These studies examined the error monitoring ability as the match between the subjective confidence ratings regarding the performance and the objective performance. Accordingly, objective performance is referred to as first-order performance, and the match between subjective confidence and objective performance is called second-order performance. This approach thus yields a categorical form of error monitoring for two-alternative forced choice (2AFC) paradigms where the performance is either accurate or inaccurate. However, in many real-life scenarios, the errors take metric forms with magnitude and direction. Thus, the 2AFC tasks in which the performance is fully categorical cannot reveal the metric characteristics of error monitoring in magnitude domains.

Recent studies have investigated metric error monitoring ability in temporal (e.g., Akdoğan and Balcı 2017; Öztel et al. 2021; Öztel & Balcı 2023a; b; Yallak and Balcı 2021; Kononowicz et al. 2019; Riemer et al. 2019), spatial (Duyan and Balcı 2020; Yallak and Balcı 2021) and numerical (Duyan and Balcı 2018;2019; Yallak and Balcı 2021) domains, where participants first estimate/reproduce (first-order task) the given target magnitude and then report their subjective confidence (second-order task). While the estimation tasks require participants to “verbally” indicate their judgments, the reproduction tasks require participants to engage in an active motor action(s) to generate a target magnitude. Finally, participants reported whether they thought their estimation/reproduction was lower or higher than the target magnitude (error direction). Overall, these studies pointed to a robust magnitude domain-specific (Yallak and Balcı 2021) metric error monitoring ability where participants could report the magnitude (indexed as lower confidence for larger errors) and direction of their trial-by-trial metric errors (for a more detailed discussion, see Öztel and Balcı 2024a).

The error monitoring ability has been widely documented to benefit from the involvement of agency in the form of motor actions in the error commitment process for 2AFC tasks (for review see Anzulewicz et al. 2019). Given that the errors committed in the reproduction tasks involve motor actions, one crucial question is to what extent temporal error monitoring is guided by actions. The involvement of motor actions in the form of agency (and sense of agency) was recently documented to have differential effects on confidence and error directionality in the timing contexts (Öztel and Balcı 2024c). Crucially, the confidence and error directionality have been found to rely on different levels of motor actions, representing the agency of the errors (i.e., self-made vs. other-made errors). Specifically, the error directionality judgments were more sensitive to the other’s errors, and confidence ratings were more sensitive to the errors that were believed to be owned (belief of agency Öztel and Balcı 2024c). This dissociation has been discussed in the sense that confidence could be relying on more introspective processes as reflecting subjective reflections on error representations. In this nature, confidence judgments could share phenomenological commonalities with the sense of agency. As a result, confidence might require a solid form of sense of agency for a correct metacognitive monitoring of the error representations (for a similar discussion, see: Oztel & Balci, 2024c). This dissociation indicates that the metacognitive information processing underlying confidence and error directionality judgments differs (Öztel and Balcı 2024c).

One of the psychophysical laws that applies to many sensory domains (e.g., brightness - Ekman 1959; stimulus size - Mckee and Welch 1992 stimulus duration - Getty, 1975) is Weber’s law, according to which the discriminability of the two physical stimuli depends on the ratio rather than the difference between two magnitudes. Weber’s law also applies to interval timing, namely the ability to time long intervals of seconds to minutes (Buhusi and Meck 2005). In conventional reproduction/production/estimation tasks, behavioral output exhibits an unimodal distribution clustered around the target duration. The scalar property of interval timing posits that the temporal estimates’ standard deviation (SD) increases proportionally to the target duration. This leads to a constant coefficient of variation (CV, which equals standard deviation (*SD*)/mean (*M*); where *M* approximates the target duration) per observer across different target durations. This uncertainty is assumed to result from the cognitive architecture of interval timing (e.g., Balcı and Simen 2016 & 2024; Gibbon et al. 1984) and becomes an integral part of the temporal representations. Relatedly, the scalar property is one way to explain Weber’s law in cognitive timing.

Many experiments demonstrated that the human timing performance conforms to Weber’s law when they decide which of the two durations is longer (e.g., Haigh et al. 2021; Grondin et al. 2001). This effect is also observed in non-human animals (e.g., mice: Öztel and Balcı 2024b; Kononowicz et al. 2022; Pigeons: Fetterman et al. 1989). Weber’s law also accounts for the numerical (e.g., Stevens and Greenbaum 1966; Teghtsoonian and Teghtsoonian 1978) and length discriminations (e.g., Stevens and Greenbaum 1966; Teghtsoonian and Teghtsoonian 1978; Crollen et al. 2013), which are considered to rely on a general metric system (Walsh 2003).

While the interval timing conforms to Weber’s law, little is known whether the temporal error monitoring ability (Akdoğan and Balcı 2017) as a form of metacognitive processing (Öztel and Balcı 2024a) relies on similar metrics of cognitive timing or sensorimotor additive noise. Only one study investigating the sense of agency (SoA) as a function of elapsed duration between events demonstrated that SoA depends on the absolute distance rather than Weber’s Ratio (WR) of the perceived durations (Erdoğan and Balcı 2024). In one sense, SoA can be considered to resemble metacognitive processing, as both require introspection regarding one’s actions relying on subjective states. Thus, this resemblance brings about the importance of testing whether similar patterns would apply to temporal error monitoring. Towards this end, we investigated (1) whether the confidence and error directionality judgments of timing performance depends on absolute error or relative timing error metrics? and (2) whether the potential metacognitive information processing differences depend on the agency of timing errors?

Accordingly, one hypothesis is that error magnitude monitoring (as indexed by subjective confidence) relies more on cognitive timing (which relies on WR) that abides to the psychophysical properties. In contrast, the error directionality judgments rely more on the motor affordances (absolute distance) that calculates the difference between the target and committed behavior without necessarily a direct access to psychophysical properties of the error representations. The prediction of this hypothesis is that confidence judgments are better predicted by the ratio of errors to the target duration and that error directionality judgments are better predicted by their absolute errors (i.e., reproduction - target).

The alternative is that, as both in Erdoğan and Balcı (2024) and Oztel and Balci (2024c), the confidence judgment relies more on absolute timing errors, whereas error directionality judgments rely more on relative timing errors. The rationale for this hypothesis comes from the fact that confidence judgments require introspective processing without necessitating any magnitude-based information processing. On the other hand, the error directionality judgments can be objectively accurate or inaccurate with potentially weaker links to introspection. This possibility is especially strong for other’s actions where the participants observe the timing behavior (Öztel and Balcı 2024a, c). This alternative predicts that confidence and error directionality judgements rely on the absolute distances and Weber’s ratio, respectively. Moreover, the effects should be more pronounced for self and other’s errors in confidence and error directionality judgements, respectively. However, it should be noted that the hypothesis of reliance to different distances is not necessarily mutually exclusive such that both error monitoring indices (i.e., confidence and error directionality) could rely on both distances to different degrees. Nevertheless, the investigation of such relative reliances carries critical importance for a deeper understanding of the psychophysical architecture of metacognitive processing in the timing domain.

In light of FAIR Data principles, we used a secondary data analysis approach to investigate our research questions without testing new participants. To this end, we used data from the first three experiments of Akdoğan and Balcı’s (2017) study (data available in the Confidence Rahnev et al. 2020) to investigate our first research question. To test the replicability of the results and investigate the second research question, we used the data from the second experiment of Öztel and Balcı (2024c). These experiments were selected for the study prior to data analyses.

## General method

Both studies aimed to investigate temporal error monitoring in adults (Öztel and Balcı 2024c: *Experiment 1*: 30 participants, 21 used in the formal data analysis; 18 female, 19 right-handed, *M*_age_ = 20.7 ± 1.63 years, one participant did not specify their age; *Experiment 2*: 21 out of 44 participants were included in the analyses, 17 female, all right-handed, *M*_age_ = 21.71 ± 4.47 years; Akdoğan and Balcı 2017; *Experiment 1*: 28 participants (24 females, *M*_age_ = 21.5 ± 3.2 years); *Experiment 2*: 24 participants (19 females, *M*_age_ = 20.9 ± 2.5 years); *Experiment 3*: 24 participants (12 females, *M*_age_ = 20.1 ± 2.0 years).

In both studies, participants were asked to reproduce a given target duration (Akdoğan and Balcı 2017*Experiment 1*: 2.1 and 4.2 s; *Experiment 2*: 1.5 and 3 s; *Experiment 3*: 3 and 6 s; Öztel and Balcı 2024c*Experiment 1* and *Experiment 2*: 1.5 and 3 s) represented with a blue square presented on the center of the screen, which varied across experimental blocks. Participants indicated their reproduction with two key presses where the first key press initiated and the second key press terminated the reproduction. As a result, the elapsed duration between the two key presses corresponded to the participants’ reproduction in every trial.

After each reproduction, participants were asked to indicate their subjective confidence regarding the proximity of their reproductions to the target duration by pressing corresponding buttons on the keyboard (1- low confidence, 2- middle confidence, 3- high confidence). After each confidence judgment, participants were asked to indicate the relative direction of their reproductions with respect to the target duration (i.e., error directionality judgment; shorter or longer than the target duration - two response options).

In Öztel and Balcı’s study (2024c), participants were asked to make similar confidence and error directionality judgements both for (a) their own reproductions (“self” condition) and (b) for another participant’s reproduction that they merely observed (“other” condition). In the latter condition, participants saw a reproduction stimulus on the screen for the duration of time as another participant’s reproduction. Thus, in the observation condition, participants did not reproduce the duration themselves. The experimental conditions were randomized across blocks. The data were collected in the lab (Akdoğan and Balcı 2017) and online (Öztel and Balcı 2024c; Pavlovia, Bridges et al. 2020) environments. Both studies were approved by the IRB and participants provided informed consent before the experiment.

## Analytical approach

For our first research question, we combined the data from the first three experiments of Akdoğan and Balcı (2017). To make data comparable with Oztel and Balci’s data (2024c) in terms of the experimental procedure, we discarded the trials from the variable blocks (i.e., the experimental blocks where the two target durations were randomized across the trials). Apart from this, we applied the same outlier exclusion criteria for all datasets. Furthermore, although not identical, sample size from all datasets were fairly comparable (included in formal analysis *N =* 21, 21, 28, 24, 24).

Before all analyses, we discarded all trials with reproduction above 3 SD from the mean reproduction per participant (representing a conservative approach to testing the temporal error monitoring performance). As a result, we removed a total of 1.8% of three sets of data from Akdoğan and Balcı (2017) and 1.1% and no data from the first two datasets from Öztel and Balcı (2024c). For the remaining trials, we calculated the signed absolute distance between each reproduction and the target duration as the following: *signed absolute distance* = reproduction-target duration and unsigned absolute distance as (unsigned absolute distance = |signed absolute distance|) and signed relative distance as (target duration - reproduction)/target duration and reproduction / target duration (resembling WR, which will be hereafter referred to as “relative distance”). Note that the two different calculations of the relative distances yielded the same BIC scores with different coefficients. For simplicity, we only report results from signed relative distance = (target duration - reproduction)/target duration as a predictor variable. The relative distance is calculated as the absolute value of the signed relative distance. Thus, the unsigned relative distance equals to |(target duration - reproduction)/target duration|.

First, separately for each dependent variable (confidence and error directionality), we fit the linear mixed effects models, where for error directionality judgments, we applied its logistic form since the statistical characteristics of error directionality judgments do not directly follow normality assumption (binary data). On the other hand, for the sake of simplicity in interpretation, we treated confidence as a continuous variable, where the ordinal ratings were linearly spaced. Note that we also provide statistical results for both error directionality and confidence as categorical predicted variables using a generalized mixed model with binomial distribution and logit link function in Supplementary Online Materials (SOM). This statistical approach accounts for the clustered data that violates the independent observations assumption of the ordinary least squares (OLS) approach for both research questions. The models are summarized in Table 1 in relation to the research questions.

**Table 1:** Linear mixed effects models that are tested for the two research questions

Does error monitoring depend on absolute error or relative error? (data: Akdoğan and Balcı 2017; Experiments 1, 2 and 3)	
Model 1a	Confidence ~ unsigned relative distance + 1|participant
Model 1b	Confidence ~ unsigned absolute distance + 1|participant
Model 2a	Error Directionality ~ signed relative distance + 1|participant
Model 2b	Error Directionality ~ signed absolute distance + 1|participant
**Do patterns of error monitoring differ based on agency? (data**: Öztel and Balcı 2024c; **Experiment 2)**	
Model 3a	Confidence ~ unsigned relative distance + 1|participant
Model 3b	Confidence ~ unsigned absolute distance + 1|participant
Model 3c	Confidence ~ unsigned relative distance*experimental condition + 1|participant
Model 3d	Confidence ~ unsigned absolute distance*experimental condition + 1|participant
Model 4a	Error Directionality ~ signed relative distance + 1|participant
Model 4b	Error Directionality ~ signed absolute distance + 1|participant
Model 4c	Error Directionality ~ signed relative distance*experimental condition + 1|participant
Model 4d	Error Directionality ~ signed absolute distance*experimental condition + 1|participant

Models were labeled in a way that numbers depict predicted variables and letters depict predictor variables in different experiments (i.e., for predicted variables; 1 and 3 for confidence respectively for Akdogan and Balci (2017) and Oztel and Balci (2024c), whereas 2 and 4 for error directionality respectively for Akdogan and Balci (2017) and Oztel and Balci (2024c). For predictor variables; ‘a’ and ‘c’ for (directional) relative and ‘b’ and ‘d’ for (directional) unsigned absolute distance)

For each model, the predictor variables were defined as fixed effects on the slope, and the participants were defined as random effects on the intercept (where “1|participants” reads as “random intercept across participants”). The continuous predictor variables were mean-centered across the cluster variable (i.e., participants in our models). All lower terms were included in the interaction models (the interaction term is depicted with “*”). We used the Restricted Maximum Likelihood (REML) method for fixed effects parameter estimates. All statistical analyses were performed in Jamovi statistical software (The jamovi project 2023).

We tested our hypotheses with a model comparison approach: we compared the Bayesian Information Criterion (BIC), which penalizes the goodness of fit index by the number of parameters that increase model complexity. This brings about lower BIC scores for better models. The ΔBIC of 2 provides positive evidence, whereas a score larger than 6 provides “very strong” evidence that the model with lower BIC scores provides better fit (Kass and Raftery 1995a, b).

## Results

### Does error monitoring depend on absolute or relative distance?

In support of temporal error monitoring, there was a significant negative relationship between the confidence judgments and the absolute value of relative and absolute distance (*p*s < 0.001). For the error directionality judgments, these effects were positive (unsigned relative distance - *p*s < 0.001). These results indicate an error monitoring ability (Akdoğan and Balcı 2017).

For the confidence judgments, the ΔBIC reveals very strong evidence favoring Model 2a over Model 1a. However, ΔBIC favored Model 1b over Model 2b for the error directionality judgements. These results suggest that confidence judgments rely more on absolute timing errors, while error directionality judgements rely more on relative timing errors. Table 1 summarizes the models and the ΔBIC information for the data gathered in Akdoğan and Balcı (2017; Experiment 1, 2, and 3). These results persisted for a generalized mixed effects model with binomial distribution and logit link function (see SOM).

**Table 2:** The model statistics for Akdoğan and Balcı (2017; data gathered from experiments 1, 2 and 3 are combined for the analyses)

Model number	ß (SE)	exp(ß)	95% CI = [Lower upper]	*p*	ΔBIC
1a	-0.56 (0.05)	—	-0.66, -0.46	< 0.001	—
1b*	-0.19 (0.02)	—	-0.22, -0.16	< 0.001	38.45
2a*	-2.87 (0.13)	0.057	0.044, 0.073,	< 0.001	42.33
2b	0.86 (0.04)	2.37	2.19, 2.57	< 0.001	—

Best models are marked with an asterisk

### Do patterns of error monitoring differ based on agency?

We first aimed to replicate our results based on a different dataset. Overall, the parameter estimates confirmed the temporal error monitoring hypotheses for both confidence and error directionality, irrespective of the predictor variable (all *p*s < 0.0001; Tables 2 and 3). Corroborating the results gathered from Akdoğan and Balcı’s (2017) dataset, the ΔBIC favored Model 3b and 4a.

**Fig. 1:**
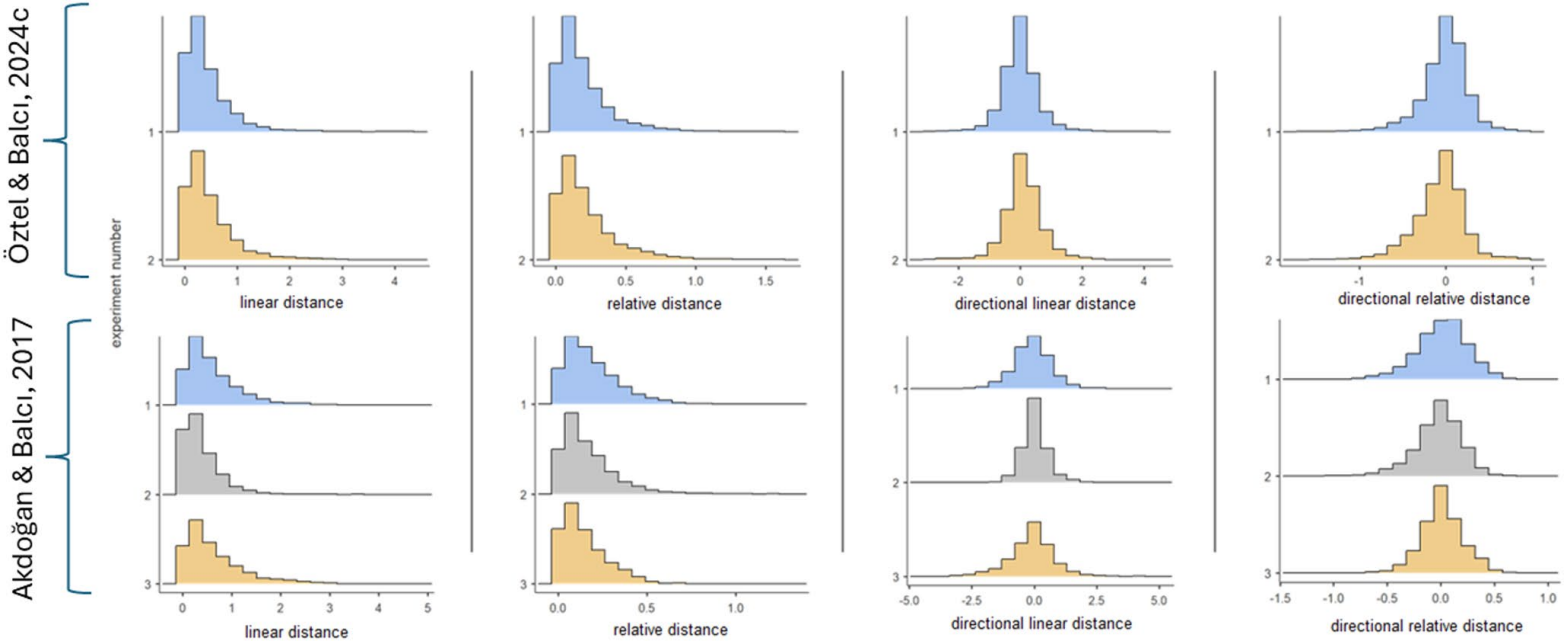
Distribution of predictor variables across different datasets and different experiments. Different rows depict different experiments in Oztel and Balci (2024c; top panel) and Akdogan and Balci (2017; bottom panel). Columns indicate different predictor variable metrics used for confidence and error directionality judgments. Across different datasets, the predictor variable distributions present similar statistical characteristics

The interaction models revealed that the participants better matched their confidence judgments to their own reproductions for both predictor variables (all *p*s < 0.0001; Tables 2 and 3). This effect was reversed for error directionality judgements, favoring the other’s reproductions (all *p*s < 0.0001; Tables 2 and 3). Table 2 presents the ΔBIC values across model pairs for Öztel and Balcı’s data (2024c; Experiment 2). Figure 1 illustrates the distributions of all outcome variables across the different datasets and different experiments. As in the case of our first research question, these results persisted for a generalized mixed effects model with binomial distribution and logit link function (see SOM).

**Table 3:** The model statistics for Öztel and Balcı (2024c; experiment 2)

Model Number	ß(SE)	exp(ß)	95% CI = [Lower Upper]	*p*	slope difference (other-self) (SE)	95% CI = [Lower Upper]	*p*	ΔBIC
3a	-0.38 (0.05)	—	-0.47, -0.28	< 0.0001	—	—	—	—
3b*	-0.21 (0.02)	—	-0.25, -0.16	< 0.0001	—	—	—	21.01
3c	-0.40 (0.05)	—	-0.50, -0.30	< 0.0001	0.39 (0.101) (both slopes are significant)	0.19 0.59	< 0.0001	—
3d*	-0.22 (0.02)	—	-0.26, -0.17	< 0.0001	0.24 (0.05) (both slopes are significant)	0.15 0.33	< 0.0001	30.89
4a*	-3.35(0.15)	0.035	0.03, 0.05	< 0.0001	—	—	—	41.16
4b	1.50(0.07)	4.48	3.93, 5.11	< 0.0001	—	—	—	—
4c*	-3.52 (0.15)	0.03	0.02, 0.04 45.51	< 0.0001	-2.69 (exp(ß) = 0.068)(0.31) (both slopes are significant)	0.04, 0.13	< 0.0001	45.09
4d	1.57(0.07)	4.79	4.18, 5.50	< 0.0001	1.17(exp(ß) = 3.21)(0.14) (both slopes are significant)	2.43 4.25	< 0.0001	—

Best models are marked with an asterisk

## Discussion

The current study tested whether the two components of temporal error monitoring (confidence rating and directionality judgment) relied on different timing error metrics (relative vs. absolute). In two distinct datasets, our results showed that the confidence judgments (as an index of magnitude monitoring) relied more on the absolute distances, which was more pronounced for owned errors. This relationship was reversed for the error direction judgments, which relied on signed relative distance, especially for others’ errors. These results align with the view that the magnitude monitoring and directionality aspects of temporal error monitoring rely on the absolute (i.e., linear) and relative (i.e., logarithmic) distances, respectively. The distinction between confidence and error directionality judgements could be due to their phenomenological differences. The confidence judgments are inherently subjective as they require an introspective performance evaluation. This view is also supported by the more robust magnitude monitoring for the owned errors (Öztel and Balcı 2024c).

The stronger relationship between confidence and absolute distance also aligns with the previous findings regarding the modulation of sense of agency (SoA) as a form of subjective experience. To this end, Erdoğan & Balci (2024) demonstrated that the strength of SoA is more strongly predicted by the absolute distance between the response and the outcome. The SoA is also measured through the ratings of subjective experiences regarding actions that potentially lead to the outcome (Erdoğan and Balci 2024). This characteristic makes SoA phenomenologically similar to confidence judgments as part of temporal error monitoring such that both SoA and confidence involve subjective attributes regarding the cognitive phenomenon associated with them. These similarities could also have behavioral (and statistical) reflections regarding their reliance on similar subjective/introspective metrics. Further, the critical involvement of motor action involving a SoA on the formation of confidence judgment has been well documented in the literature (for a similar discussion, see: Anzulewicz et al. 2019). Indeed, the unsigned absolute distance that is driven by motor actions (informed by the internal feedback mechanism) predicts confidence judgments more strongly, which is in support of the view that confidence ratings and SoA share phenomenological similarities. Thus, this result serves as further support for the phenomenological commonalities between confidence and SoA as reflected in the shared psychophysical characteristics.

The relationship between the motor responses and the metacognitive performance that is indexed with confidence judgements is also documented in the perceptual domain. For example, one study found that although the metacognitive accuracy was above the chance level in both prospective and retrospective judgments, it was lower when confidence judgements were given before (i.e., prospectively) than after the first-order memory judgment (i.e., retrospectively; Siedlecka et al. 2016). Similarly, the speed of self-generated motor actions was predictive of confidence judgments associated with perceptual judgements (Palser et al. 2018); slower perceptual judgments were more accurately monitored metacognitively than the faster perceptual judgments, demonstrating that the action speed may indeed guide the metacognitive accuracy (Palser et al. 2018). Together, these findings demonstrate the contribution of the motor signals (at least as a proxy) to metacognitive performance.

The distinct phenomenological feature of error directionality judgments can be attributed to the fact that there is objective accuracy associated with these judgements; the temporal reproductions are either shorter or longer than the target duration. This brings about an additional characteristic that covers the objective accuracy aspect for the error directionality judgements. This aspect could be rendering the error directionality judgments phenomenologically similar to the first-order processing during temporal comparison tasks (see also Öztel and Balcı 2024a, c), where participants report which of the two durations is longer (e.g., Vicario et al. 2008).

The resemblance between the error directionality judgment and the first-order processing in the temporal comparison tasks can account for the stronger predictive power of logarithmic than the unsigned absolute distance between the reproduction and the target. Especially for the unowned timing errors (i.e., “other” condition in Öztel and Balcı 2024c), this phenomenon is more pronounced where the introspective processing is irrelevant due to the absence of motor signals. The better error direction monitoring for the other’s errors provides further support for this view (Öztel and Balcı 2024c).

Taken together, the current results further validate the distinctive features of confidence and error directionality judgments as previously described in Öztel and Balcı (2024c; see also Öztel and Balcı 2024a): the error directionality judgments abide by Weber’s law, whereas the confidence judgments that index the magnitude of timing errors relies on the internal signals elicited via the motor actions. Thus, the current study confirms the idea that where applicable, the error magnitude and directionality should be separately investigated in metric error monitoring studies (as opposed to the composite score approach, which unifies error magnitude and directionality, e.g., as in Akdoğan and Balcı 2017).

Note that these results do not point out that the two components of temporal error monitoring (i.e., the confidence and error directionality) are fully dissociated. If this were the case, one would observe mutually exclusive predictive power for the absolute and logarithmic distances on the confidence and error directionality judgments. Instead, current results indicate that both temporal error monitoring components can be predicted by different metrics to different degrees. Thus, while highlighting the distinctive phenomenological features, the current results also validate the commonalities of confidence and error directionality judgments, stemming from the subjective processing of owned actions. These commonalities could be explained by the real time readings of the two independent clocks for the objective/reference and subjective/motor time, in which these readings are solely based on the absolute-online distances between the two clock states. This model allows timing behavior to be fine tuned based on threshold adaptation in accordance with the first threshold crossing clock (Balci and Oztel 2025). While this kind of an adaptive threshold model describes the emergence of the confidence and error directionality judgment as stemming from a single real time readings of temporal information, the current study, along with Oztel and Balci (2024c) show that these two error monitoring metrics are at least partially dependent on distinct phenomenological and computational processes. Future studies could address this possibility at the computational level by testing if the relative-online distances (i.e., ratio of real time unsigned absolute distance to the cumulative average of unsigned absolute distance) could also predict confidence and error directionality judgments for the prospective fine tuning of the timing behavior. In addition, future research should investigate these commonalities and distinctive features at the neural level.

There are several limitations associated with the current study. First, the current study involves a secondary data approach, which limits its scope for establishing a causal link between the different error types (i.e., absolute or relative distances). Although the datasets that we chose for the current study fairly matched in terms of experimental procedure and participant demography, we could not directly match all procedural details and sample characteristics. Furthermore, since the hypotheses that we tested were not mutually exclusive, the obtained results in the current study cannot directly describe the idiosyncratic features of confidence and error directionality judgments in the temporal error monitoring context. Future studies should address these idiosyncratic features at both neural and computational levels.

## Conclusion

The current results show that confidence and error directionality judgments have distinctive phenomenological features. The confidence judgments rely more on the unsigned absolute distance between the reproduction and the target duration; which can be referred to as the errors in the motor timing. The error directionality judgments, similar to the first-order temporal discrimination (e.g., Haigh et al. 2021; Grondin et al. 2001), rely more on relative distance. These reliances are even more pronounced for owned and other’s temporal errors, respectively. Finally, the predictive feature of both of these distances regarding the two temporal error monitoring components also highlights the partial commonalities of these two components.

## Supplementary Information

Below is the link to the electronic supplementary material.

Supplementary Material 1

## Data Availability

The data and materials are available at Open Science Framework (OSF; Öztel and Balcı 2024c: https://osf.io/mycza/?view_only=4513829013834aba98fb0c1d2ab6062d) and Confidence Database (Rahnev et al. 2020: https://osf.io/s46pr/; corresponding author should be contacted for the materials of Akdoğan and Balcı 2017).

## Code availability

No coding was conducted for analyzing the data of the current study.
