# Supplementary material for: Psychophysical dissection of temporal error monitoring
Source: Cogn Process. 2025 Sep 30;27(1):157–65. doi: 10.1007/s10339-025-01302-8 (PMC12860838; doi:10.1007/s10339-025-01302-8)
Supplement: Supplementary file 1 — Supplementary Material 1 [file 10339_2025_1302_MOESM1_ESM.docx]

**Supplementary Online Materials**

| **Model Number** | **ß (SE)** | **95% CI = [Lower Upper]** | ***p*** | Δ**BIC** |
| --- | --- | --- | --- | --- |
| 1a | -2.67 (0.24) | -3.14, -2.2 | < 0.001 | — |
| 1b* | -0.875 (0.072) | -1.02, -0.734 | < 0.001 | 22.826 |
| 2a* | -2.868 (0.13) | -3.123, -2.61 | < 0.001 | 15.333 |
| 2b | 0.863 (0.041) | 0.783, 0.943 | < 0.001 | — |

*Table S1.* The model statistics for Akdoğan and Balcı (2017, data gathered from Experiments 1, 2 and 3 are combined for the analyses). Best models are marked with an asterisk. For both predicted variables (i.e., confidence and error directionality) the generalized linear mixed effects model was fit with a binomial distribution and a logit link function.

| **Model Number** | **ß(*SE*)** | **95% CI = [Lower Upper]** | ***p*** | **slope difference (other-self) (*SE*)** | **95% CI = [Lower Upper]** | ***p*** | **ΔBIC** |
| --- | --- | --- | --- | --- | --- | --- | --- |
| 3a | -1.74 (0.163) | -2.06, -1.42 | <0.001 | — | — | — | — |
| 3b* | -0.83 (0.075) | -0.979,-0.684 | <0.001 | — | — | — | 7.626 |
| 3c | -1.81 (0.17) | -2.13, -1.48 | <0.001 | 1.17 (0.35)  (both slopes are significant) | 0.48,1.85 | <0.0001 | — |
| 3d* | -0.87 (0.077) | -1.02, -0.72 | <0.0001 | 0.71 (0.16)  (both slopes are significant) | 0.40, 1.03 | <0.0001 | 17.298 |
| 4a* | -3.35(0.15) | -3.64, -3.07 | <0.0001 | — | — | — | 41.693 |
| 4b | -3.35 (0.15) | -3.64, -3.07 | <0.0001 | — | — | — | — |
| 4c* | -3.51(0.15) | -3.82, -3.21 | <0.0001 | -2.69(0.31)(both slopes are significant) | -3.31, -2.08 | <0.0001 | 45.093 |
| 4d | 1.57(0.07) | 1.43, 1.71 | <0.0001 | 1.17(0.14) (both slopes are significant) | 0.89,1.45 | <0.0001 | — |

*Table S2.* The model statistics for Öztel & Balcı (2024c; Experiment 2). Best models are marked with an asterisk. For both predicted variables (i.e., confidence and error directionality) the generalized linear mixed effects model was fit with a binomial distribution and a logit link function.
